# A new perspective on Workload Control by measuring operating performances through an economic valorization

**DOI:** 10.1038/s41598-022-17968-5

**Published:** 2022-08-26

**Authors:** Davide Mezzogori, Giovanni Romagnoli, Francesco Zammori

**Affiliations:** 1 Department of Engineering “Enzo Ferrari”, University of Modena and Reggio Emilia, Via P. Vivarelli, 10, 41125 Modena, MO Italy; 2 Department of Engineering and Architecture, University of Parma, Viale delle Scienze, 181/A, 43124 Parma, PR Italy

**Keywords:** Electrical and electronic engineering, Mechanical engineering, Statistics, Computational science

## Abstract

Workload Control (WLC) is a production planning and control system conceived to reduce queuing times of job-shop systems, and to offer a solution to the lead time syndrome; a critical issue that often bewilders make-to-order manufacturers. Nowadays, advantages of WLC are unanimously acknowledged, but real successful stories are still limited. This paper starts from the lack of a consistent way to assess performance of WLC, an important burden for its acceptance in the industry. As researchers often put more focus on the performance measures that better confirm their hypotheses, many measures, related to different WLC features, have emerged over years. However, this excess of measures may even mislead practitioners, in the evaluation of alternative production planning and control systems. To close this gap, we propose quantifying the main benefit of WLC in economic terms, as this is the easiest, and probably only way, to compare different and even conflicting performance measures. Costs and incomes are identified and used to develop an overall economic measure that can be used to evaluate, or even to fine tune, the operating features of WLC. The quality of our approach is finally demonstrated via simulation, considering the 6-machines job-shop scenario typically adopted as benchmark in technical literature.

## Introduction

Nowadays, more than 40 years after its original conception, Workload Control (WLC) has been deeply investigated^1^ and its manyfold benefits are unanimously acknowledged by the scientific community^2^. Nonetheless, although they have increased over the last decade, successful industrial implementations of WLC are still limited^3–5^. A possible explanation of such a limited industrial consideration could be the complexity and the heavy informational requirements of WLC, which are both higher than those of other ‘paper and pencil’ lean-techniques, such as dual Kanban or Constant Work In Progress-ConWIP^6^. Another hurdle to WLC acceptance can be found in^7,8^, where the large gap between theoretical contributions and industrial applications is clearly discussed and exemplified. Both authors noted that most of the studies are carried on using over-simplified event-based simulation models, which do not account for real-life constraints. Perfectly balanced systems with a rather low utilization level (typically set at 90%) are generally considered, while additional operative issues such as handling times, limited buffer areas and parts-feeding requirements are neglected^9^. Although the spectrum of the business cases is much wider than that^10^, only a few researchers focused on unbalanced manufacturing systems (see for example^11^) and tried to investigate the applicability of WLC to these system too^8,12^.

A last and more subtle problem has been identified by^6^, who noted that in scientific literature WLC performance is generally measured using time-related measures, such as the percentage of tardy jobs or the like. There is no doubt that the capability to set and respect short delivery dates in response to customers’ enquiries is a primary market requirement for Make-To-Order (MTO) manufacturers^13,14^. Yet, focusing exclusively on this measure is somehow myopic because, if WLC is regulated by simple operating rules, the percentage of on time deliveries does not deviate much from that of a purely push operating system, with optimized dispatching rules^15,16^. This is a strong disincentive, as manufacturers do not see the point of adopting a complex production planning and control (PPC) system, if similar results could be achieved with a much simpler MRP based system, provided of adequate capacity control and scheduling mechanisms^17,18^. To get rid of this misconception, the research community should extend the analysis beyond ‘time related’ performance measures, and should try to quantify other WLC related benefits, such as WIP reduction and containment. WIP, in fact, is the worst cause of waste of a lean-oriented manufacturing systems and its reduction, beside containing the shop floor throughput time, would also cut holding costs, free up space, improve order and tidiness, and favor visual management and control^19^. As noted by^4^, performance measures related to these elements of competitive edge exist, but they are seldom used to assess performance of WLC systems. Also, these are unstructured approaches that focus on a single and specific feature of a WLC systems, so that the overall view, and the overall benefits are lost anyway.

For this reason, an interesting branch of research has developed over the last decade, with the aim of evaluating WLC performances by means of economic values, that is costs and/or revenues^20–22^. Although this research topic is also reflected in the wider literature on weighted earliness and tardiness (see for example^23^), we believe that an economic evaluation in monetary terms is the main way to merge and combine different benefits and costs. To this aim, all the relevant costs (of variable nature) and incomes of a specific WLC configuration might be quantified, confronted, and discussed, to assess and compare the expected economic benefits of the WLC implementation.

However, the mentioned studies on economic assessment of WLC performances present some important limitations from our point of view. First, the cost system they propose are specific of the simulated case^22,24,25^, and often industry-specific and related to semiconductor manufacturing^20,26–28^. Second, although the cost factors, or clearing functions, have been reported in previous research, we note that these methods were seldom adopted to the so-called research field of rule-based WLC (see^29,30^ for an overview on rule-based and optimization based WLC).

Indeed, we believe that such a *system*, as well as the cost function, should be better generalized and detailed, especially in the rule-based WLC field. We also note that previous research mostly focus on costs, whereas we believe that incomes should also be considered to provide a better performance indicator. Finally, in the authors’ opinion, previous research works do not clearly report how an economic indicator could be used to identify and fine-tune a specific WLC configuration, and it could also provide to companies, and especially SMEs, a method to assess economic benefits to be expected from a WLC implementation.

To overcome these limitations, the present paper incorporates a general method for economic evaluation in WLC research, by proposing a Revenue per job function that could be used to select and fine-tune the best performing WLC configuration in given circumstances. This function is also applied to a simulated 6-machines job-shop scenario, which is typically used as a benchmark in technical literature, and supported by a sensitivity analysis, so as to show the benefits of our approach in a common scenario, and to assess how these benefits might change under different ratios of cost parameters. By doing this, we suggest a possible solution to the problem identified by^6^, i.e. we provide a general method, as well as a sample implementation and a sensitivity analysis to identify a trade-off between different performances and metrics.

The remainder of the paper is organized as follows. In “An overview on WLC research” and “Performance assessment of a WLC system” sections we provide a brief overview of WLC research, also including studies on performance assessment of WLC systems. Moving from these premises, the economic assessment model is developed in “A proposal for an economic evaluation of WLC systems”, where all the cost and revenues voices are discussed in detail. The approach is finally tested via discrete event simulation in “Performance assessment of a WLC system” and Performance assessment in monetary terms” sections that describe, respectively, the simulated environment and the obtained outcomes. Comments, managerial insights, and conclusions are finally provided in “Conclusions, managerial implications, and future works”.

## An overview on WLC research

A major guiding principle of WLC is input/output control^31^, asserting that the input and the output rate of a shop should equal. Scientific literature, however, presents several WLC methods, whose unifying theme is use of a pre-shop pool and order release mechanism^1^. In this paper, we refer to the so-called LUMS WLC (see for more details^32^), a rule-based approach to WLC consisting of three (or four) control levels, namely the (i) customer enquiry and order entry, which are sometimes separated in two different stages; (ii) order release and (iii) priority dispatching. At each level, two methods of control are available: input and output control^33^. The former regulates the input flow of orders to the next stage, and the latter controls workload by regulating the outward flow, e.g. by setting due dates and adjusting capacity. In this regard, we note that WLC is partially aligned with the stream of research on Value-Chain Flexibility^34–36^. According to this framework, in fact, to answer to the environmental uncertainty that is typical of job shops oriented manufacturers, and which has significantly increased in the post-pandemic era, organizations should exploit four different types of flexibility, namely: (i) product development; (ii) manufacturing; (iii) logistics and (iv) spanning flexibility^37^.

The alignment between these two concepts lies in the fact that WLC could significantly impact on manufacturing flexibility, and especially on machine and routing flexibility (see^37^), and it could also provide some positive outcomes on other types of flexibility.

To improve flexibility and stabilize lead times in a context of environmental uncertainty, WLC uses workload caps or norms, generally expressed as number of expected working hours, to regulate the release of new production orders. More precisely, work in process is constantly monitored and new jobs are not released in the shop floor, unless all norms are fully met. If one or more norms are violated, jobs are stopped into a Pre-Shop-Pool (PSP) of pending orders, waiting to be reconsidered for possible future release. Due to this pattern, the time a job spends in the PSP, namely the ${\text{PSP}}_{{{\text{time}}}}$, may not be negligible relatively to its Shop Floor Throughput Time (${\text{SFTT}}$), i.e., total time from order release to dispatching. Hence, in a WLC system, these two quantities are always considered together as they determine the Gross Throughput Time of a job ${\text{GTT}} = \left( {{\text{PSP}}_{{{\text{time}}}} + {\text{SFTT}}} \right)$.

When a customer places an order (or makes an order enquiry), the manufacturer must decide whether to accept the order or not. Only profitable and technically feasible jobs should enter the PSP. Since this evaluation step is standard and typical of many manufacturing system, it is rarely considered by the WLC research community. A more interesting decision, which has sparked much more research interest, concerns the customer enquiry stage and the assignment of a due date to the accepted jobs^38^. Such choice, which often requires a bargain process with the customer, may also impact on the acceptance decision because, if the required due date is too thigh, the manufacturer could incur into penalties, and could even jeopardize the respect of the overall production plan, by trying to preempt a critical job, to speed up its delivery. Hence, customer enquiry stage and order acceptance are generally treated in parallel.

The job release phase is the crucial part of a WLC system. The adopted technique for selecting and releasing orders from the PSP to the shop floor can make the difference between a successful and unsuccessful WLC implementation^3^. Specifically, all jobs pending in the PSP are periodically considered for possible release to the shop floor. This task is performed in sequence, by considering one job at a time, from the first to the last one in the PSP. In the PSP, indeed, jobs are sorted in order of priority, typically by means of a dispatching rule, which is usually the same used to sort jobs in the shop floor. Release depends both on the workload of the considered job and on the current system’s state (expressed as the level of the norms), as detailed below.

Let $w_{j,m}$ be the workload of job $j$ on machine $m$ and let $\tilde{j}$ be one of the job in the PSP. When, at time $t$, $\tilde{j}$ is selected for possible release, its workload $w_{{\tilde{j},m}}$ is used to update the cumulative workload released to the system ($W_{t}$) and/or that released to a specific machine $(W_{m,t} )$.

The update is done as in Eqs. (1) and (2), whereas the cumulative workloads are computed as in Eqs. (3) and (4), respectively.

1 $$W_{{m,t^{ + } }} = { }W_{m,t} + w_{{\tilde{j},m}}$$

2 $$W_{{t^{ + } }} = W_{t} + \mathop \sum \limits_{m} w_{{\tilde{j},m}}$$

3 $$W_{m,t} { } = { }\mathop \sum \limits_{{j \in J_{{\left( {S,m} \right)}} { }}} w_{j,m}$$

4 $$W_{t} = { }\mathop \sum \limits_{m} \mathop \sum \limits_{{j \in J_{{\left( {S,m} \right)}} }} w_{j,m} = \mathop \sum \limits_{m} W_{m}$$

where $t^{ + }$ is the time immediately after job $\tilde{j}$ has been selected for possible release, $J_{{\left( {S,m} \right)}}$ is the subset of jobs released in the shop floor, which still need to visit machine *m*, $w_{{\tilde{j},m}}$ equals zero for all machines *m* that does not lay on the routing of job $\tilde{j}$.

It is worth noting that these equations are used at different times. Whereas Eqs. (1) and (2) are related to the selection process, Eqs. (3) and (4) are triggered by the manufacturing processes in the shop floor. Equations (1) and (2), in fact, are evaluated any time a job of the PSP is select for possible release, whereas Eqs. (3) and (4) are applied every time a job in the shop floor moves from a machine to the next one.

We also note that, if a single norm is used to constrain the cumulative workload of the system $W_{t}$, the release phase is named ‘total shop load’ control. Conversely, if multiple norms are used to limit the cumulative workload $W_{m,t}$ of individual machines, the release phase is labelled as ‘bottleneck load’ or ‘load at each machine’, if control is limited to the supposed bottlenecks or extended to all machines, respectively (for more details, see^39–41^).

Anyhow, the updated values $W_{{t^{ + } }}$ or $W_{{m,t^{ + } }}$ are compared to the system’s norms and the job $\tilde{j}$ is released to the shop floor, provided that none of the norms is violated. If not so, $\tilde{j}$ remains in the PSP and the process is iterated until the whole list of jobs in the PSP has been completed.

Relatively to the release phase, another crucial decision regards the quantification of the individual workloads $w_{j,m}$ of the entering jobs. Generally speaking, the workload released to a certain machine $m$ can be split into a direct and an indirect part. The first part is related to the jobs that are in the queue of machine $m$; this part is considered ‘direct’ because these jobs are currently using the productive capacity of machine $m$. The second part, instead, is due to the jobs that are still upstream of machine $m$ and that will visit this machine later on. This part of the workload is considered ‘indirect’ because these jobs will use the productive capacity of machine $m$ only in a later moment. Hence, the workload $w_{j,m}$ should be fully included in $W_{m}$ if job $j$ is queuing at machine *m* and should be partially included in $W_{m}$ if job $j$ is still upstream of *m*. In other words, $w_{j,m}$ should be rescaled (or reduced) to consider the time lag that will elapse before $j$ reaches $m$: the more $m$ is downstream, the fewer load should be attributed to it ^42^. How to properly rescale the workload $w_{j,m}$ is a highly researched topic. A first attempt, known as the Load Oriented Order Release (LOOR) approach, was proposed by ^43^, who dynamically rescales $w_{j,m}$ using a depreciation factor obtained through a statistical (mainly regression based) analysis. Other probabilistic approaches have been proposed next as in^44,45^. Also, a specific methodology was developed at Lancaster University Management School, also referred to as LUMS methodology, designed for the specific needs of MTO companies and concentrated upon the Aggregate Load field of WLC. However, despite the research interest that they roused, these approaches have seldom found their way into practice and nowadays they are increasingly less used in the literature^46^. As^47^ pointed out, in fact, sophisticated methods are too complex for a straightforward application in the industry and, consequently, they have been misused for lack of understanding and neglected over time. To solve this operational problem, other authors (see for example^48^) simplified the problem by considering $w_{j,m}$ as a fixed value equal to the sum of the processing and set up time of job *j* on machine *m*. This approach is known as the ‘aggregate approach’, since the direct and indirect part of the workload are not differentiated and $w_{j,m}$ is never rescaled, not even if job $j$ is queuing at a machine upstream of $m$. A more refined alternative, known as the ‘corrected aggregate approach’, is frequently applied too.

According to this approach, introduced by ^33^, the workload is rescaled as in Eq. (5):

5 $$\tilde{w}_{j,m} = \frac{{w_{j,m} }}{{n_{j,m} }}$$

As Eq. (5) shows, the rescaling rule is very simple, as the corrected load $\tilde{w}_{j,m}$ is obtained using as scaling factor the position $n_{j,m}$ of machine $m$ in the routing of job $j$. Like the ‘aggregated approach’, the load is rescaled as soon as job $j$ is released and, next, the value of $\tilde{w}_{j,m}$ will no longer be changed. The use of the corrected aggregate approach is generally preferable, as it assures high performance, and it also simplifies the fine-tuning of the norms, as described in greater details by^15,41^.

Finally, jobs in the shop floor are managed as in a standard batch and queue system, i.e. they are pushed from a machine to the next one. For this reason WLC is generally considered a hybrid push–pull technique. Hence, when more jobs compete for the same machine, WLC uses some predefined dispatching rule. However, if norms have been properly fine-tuned, queues tend to be short and stable and so jobs dispatching become a less important issue that can be simply treated using one of the manyfold dispatching rules proposed in the literature^49^. For a comprehensive discussion of the most common dispatching rules we refer the interested reader to^50^.

## Performance assessment of a WLC system

As already mentioned, WLC assessment is frequently carried on using tardiness and/or percentage of tardy jobs as performance indicator. However, some examples of alternative measures can be found in the literature, as exemplified in Table 1, which shows the most common measures grouped in the broad categories suggested by^4^. We note that, with respect to^4^, the internal co-ordination category is not reported. To the best of the authors knowledge, in fact, such measures (e.g. co-ordination between production and marketing) have not been used in the last decade.

**Table 1:** Performance measures used in recent scientific works.

Category	Measures	Sample references
1. Time-related	Lead time	^51^
	Shop floor throughput time	^52^
	Supply chain time	^53^
2. Dependability	Lateness	^54^
	Percentage of tardy jobs	^15^
	Service level	^25^
	Tardiness	^32^
3. Shop load-related	Number of jobs in queue	^55^
	Utilization rate	^56^
	WIP level	^8^
	Workload balance	^57^
4. Market-related	Percentage of accepted orders or ‘strike rate’	^58^
	Volume of accepted orders	^59^
5. Cost-related	Inventory level (WIP and Finished goods)	^30^
	Production yield	^58^
	Comprehensive cost measure	^22,30^

Almost all the metrics reported in Table 1 are self-explaining; only some of them deserve some further comments. *Supply chain time* was introduced by^53^ to measure lead time of a MTO supply chain of highly customizable goods. Also^58^, uses two interesting measures, namely the *Strike rate*, that is the proportion of successful quotations among total enquiries, and the *Production yield*, referred to as the successful rate of the production of a product, or the percentage of jobs that do not generate quality problems, such as reworks or scraps.

It is important to observe that most of the studies makes use of ‘time related’, ‘dependability’ and ‘shop load-related’ measures, with the first two categories being the most widely used^6^. ‘Market-related’ measures are, indeed, less common: the table reports^59^, for instance, which compared alternative production planning and control systems in terms of the generated volumes (and monetary value) of the accepted orders; and^58^, where the information architecture for WLC is detailed, reporting a case study of a small precision-engineering MTO manufacturer.

Although the set of available measures is rather ample and variegated, a common and standard way to assess real-word performance of WLC and, more in general, of a PPC system, is still missing. Researchers, in fact, are often tempted to report the measures that better confirm their hypotheses, often arguing that those performance measures are the most pertinent in the industrial case under analysis. Consequently^4^, suggested that researchers should “ask themselves whether the same set of measures should be used to assess WLC implementation in practice, in order to determine appropriate measures for their project”. Unfortunately, despite this suggestion, many scholars still continue to use very specific measures that might fail to capture the overall benefits of a PPC system, also due to the fact that some of these measures are conflicting (e.g. short lead time versus high service level, or high volume of orders versus small percentages of tardy jobs) a fact that makes extremely hard the definition of adequate benchmarks. The need, and the relevant practical implications, of a standardized measure is evident, but little has been done so far, toward this goal.

A recent stream of research has indeed started to investigate WLC performances from a more comprehensive point of view. A preliminary single-machine cost function was proposed in 1997 by^60^, by calculating a weighted sum of the WIP and tardiness costs under a periodic order release policy. The first cost function that could be applied to a WLC system, however, can be found in the influential work of Asmundsson et al*.*^61^, proposing the clearing function models, which are deemed as advanced and widely used models to date in terms of optimization-based order release models^30^. In 2011^62^, jointly considered different and potentially conflicting performance measures, by converting them in monetary terms. The same approach was followed by^63^, who compared alternative production plans considering three different kind of costs: (i) WIP holding costs, (ii) finished goods holding cost, and (iii) backlog cost. Within this stream of research^28^, reported an interesting experimental comparison of different iterative approaches and a clearing function, estimated by means of two different approaches, in a simplified model of a wafer fabrication facility. One year after^27^, implemented two linear programming models for cycle time estimate and presented the computational testing of a production planning model based on clearing functions on a large-scale data set that represents a wafer fabrication facility with two products. The performances of different plans are then compared based on the profit realized by the facility when the execution of the plans is simulated. Reference^26^ assessed and compared, under a wide range of operating conditions, the performances of a production planning model with fixed lead times and a second model with workload-dependent lead times in a rolling horizon setting.

Another emerging stream of research has made use of adaptive planning system, such as machine learning algorithms and artificial neural networks, to dynamically set lead times, plan capacities and decide on whether to release orders or not^15,24,25,41^. Finally, starting from the seminal work^29^, interesting studies have been carried out in the very last years to bring together and compare the rule-based WLC, i.e. order release mechanisms determining the release times of the orders by means of a set of rules, and optimization-based WLC, i.e. multi-period order release mechanisms that use optimization models^20,22,30^.

While we praise the effort of these authors, we also note that:

- To the best of our knowledge, an economic valorization has seldom been used in rule-based WLC literature;
- Relative to such a WLC scenario, the cost structures proposed are not complete, as they do not account either for production costs or for sales prices, thus missing the huge difference between accepted and non-accepted customer’s orders;
- A general method for economic evaluation of WLC systems is still missing in rule-based WLC literature.

The approach to properly evaluate WLC based upon economic considerations is the topic of the following sections.

## A proposal for an economic evaluation of WLC systems

Our proposal is to evaluate performances of different WLC systems, by considering incomes and costs items that closely match the classification structure provided in Table 1. All proposed costs and incomes are traditionally used in inventory management theory and can be easily estimated and measured in production systems^64^. If a Manufacturing Execution System is in place, all the inputs of the income/cost equations (provided next), can be easily collected on the field. Should this not be the case, the standard costs and the standard times reported, respectively, in the bill of materials and in the production-cycle can be used as a viable alternative. Similarly, if a simulation model is needed, these costs can be easily embedded in the simulation logic and used to fine tune the norms and to operate the system at the point of maximum economic viability.

### Time and shop load related costs

The first category contains two costs items related to the time a job has spent in the shop floor, either being processed or waiting in queue. These are the Transformation ($TR$) and WIP Holding costs ($WH$), respectively. Concerning $TR$, it includes direct materials costs, all direct production costs (sustained on the shop floor) and, eventually, the setup costs, as shown in Eq. (6).

6 $$TR_{j} \, = \,Direct\,\,Materials\, + \,Processing\,\,~Cost\, + \,Setup~\,\,Cost\, = \,{\mathcal{M}}_{j}  + \mathop \sum \limits_{m} \left( {t_{{j,m}} p_{m}  + \frac{{\mathcalligra{t}_{{j,m}} s_{m} }}{{b_{j} }}} \right) $$

where ${\mathcal{M}}_{j}$ is the total cost of the direct materials (or parts, or components) used to manufacture job $j$, $t_{j,m}$ is the actual processing time of job $j$ on machine $m$, $p_{m}$ is the cost of use per unit of time of machine $m$, which should include depreciation, energy, direct labor and, eventually, auxiliary operating costs, $\mathcalligra{t}_{{j,m}}$ ﻿is the actual setup time of job $j$ on machine $m$, $b_{j}$ is the batch size of job $j$, $s_{m}$ is the setup cost per unit of time.

If needed, the handling costs related to the distance covered by a job along its route could be included in Eq. (6) too. We decided not to do so, because handling costs (as well as other cost items of logistic nature) depend on the layout of the system but not on the way in which a WLC system has been dimensioned and fine-tuned. The same would not be true if the aim were to compare alternative PPC systems that leverage on specific handling and/or feeding systems such, for example, as a dual Kanban system with milk-run feeding strategy^65^. In this case, indeed, handling costs would change with the considered alternative. Please note that also the transformation costs herein considered do not depend on the adopted WLC system; yet a precise quantification of these costs is essential for a correct valorization of WIP and Stocks that, instead, are highly influenced by the WLC system in use.

Concerning $WH$, this item accounts for the cost sustained to hold work in process in the shop floor and it is obtained by multiplying the total time in the system, or Shop Floor Throughput Time ($SFTT$), for the unitary holding cost per unit of time $h$. That is:

7 $$WH_{j} \, = \,Time\, \,in \,\,the \,\,sistem \cdot Unit\,\,Holding\,\,Cost\,\,per\,\, unit\,\,of\,\, time\, = \,\left( {SFTT_{j} \cdot h_{j} } \right)$$

The holding cost $h_{j}$ depends on the value of job $j$ and, therefore, it increases as job $j$ moves downstream its manufacturing process. Also, assuming that materials are evenly added throughout the manufacturing process (as for an assembly line) and that the cost of use $p_{m}$ does not vary much from one machine to another, the increase of $h_{j}$ can be considered linear. So, the holding cost can be expressed as in Eq. (8):

8 $$h_{j} = k_{h} \cdot \left( {0.5 \cdot TR_{j} } \right)$$

where $k_{h}$ is a fixed coefficient lower that one, and $0.5TR_{j}$ is the average value of job $j$.

If all materials enter at the beginning of the production cycle, the computation of the holding cost changes as in Eq. (9):

9 $$h_{j}  = k_{h}  \cdot \left( {{\mathcal{M}}_{j}  + 0.5 \cdot \mathop \sum \limits_{m} \left( {t_{{j,m}} p_{m}  + \frac{{\mathcalligra{t}_{{j,m}} s_{m} }}{{b_{j} }}} \right)} \right)$$

### Dependability related costs

This category contains the costs incurred by the company to meet customers’ needs in terms of delivery times. Two items are considered, namely the Storage costs ($ST$) of end goods and the Penalty costs ($PN$).

The first item, related to earliness, accounts for the fact that orders completed on time must remain in stock for some time before being finally aggregated with other orders and dispatched to the end customer. So, expenses for storage of the manufactured items are incurred. The second item, instead, is related to tardiness and is incurred anytime orders are shipped late. Note that penalties may be explicitly agreed with the customer at the contractual level, but even if this were not the case, an implicit cost should be considered, at least to cover for backlog issues such as: customer compensation for delay, loss of goodwill, expedited shipping, potential loss of future orders, as well as for other indirect costs. Also note that storage and penalty costs can be considered as mutually exclusive, as either one or the other must be null for every order. Indeed, it can be assumed that late orders are never held in stock (or are held for a very short time) but are shipped immediately.

Operatively, akin the holding cost for WIP, also the holding cost of the finished goods are obtained by multiplying the time spent by a job in the finished goods inventory for its monetary value. Since jobs have been completed and are ready to be delivered, their value coincides with the total transformation costs. So, we have that:

10 $$ST_{j} \,\, = \,Inventory\,\,Time \cdot Unit~\,\,Holding\,\,~Cost~\,\,per\,\,~unit~\,\,of\,\,~time = \,\left( {IT_{j}  \cdot {\mathcalligra{h}}_{j} } \right) = IT_{j} h\left( {k_{h}  \cdot TR_{j} } \right)$$

where $IT_{j}$ is the total inventory time and the unit holding cost ${\mathcalligra{h}}$ is again obtained as a fraction of the total transformation cost $TR_{j}$.

Penalties costs are harder to be estimated, and this topic has been intensively studied in the Make-To-Stock sector, whose literature on Out-Of-Stock could provide some interesting suggestions to the reader (see for example^66,67^ for quantitative studies on Out-Of-Stock in the fast-moving consumer goods sector). Yet, neglecting costs of penalties would lead to an inaccurate cost structure. So, our suggestion is to calculate $PN$ by multiplying a unitary penalty cost (or fee) per unit of time, say $f$, for the total delay $D$, expressed as the positive difference between the shipping time $ST$ and the due date $DD$ agreed upon with the customer. So, we have:

11 $$PN_{j} \, = \,Total \,\,Delay\, \cdot \,Unit\,\,Penalty\,\, Cost \,\,per \,\,unit \,\,of\,\, time\, = \,\left( {D_{j} \cdot f_{j} } \right)\, = \,k_{p} \cdot P_{j} \cdot {\text{max}}\left\{ {0,\left( {ST_{j} - DD_{j} } \right)} \right\}$$

where in analogy with the holding cost, the coefficient $k_{p}$ is used to express the penalty $f$ as a fraction of the sale price $P$, per unit of delay time. We note that it could be wise to limit the penalty cost to a maximum value, such as a given percentage of the sales price, to rule out the risk of unrealistically higher penalty costs due to very long delays.

### Market related items

For a fair comparison, the generated turnover should be included in the analysis, too. Different PPC systems, in fact, may lead to a different throughput rate and, consequently, to non-negligible changes in the production volumes and turnover. Note that this issue is even more stringent when WLC is considered, as different ‘job acceptance’ rules may modify not only the productive volumes, but also the productive mix, with strong impact on the overall profitability.

In case of MTO companies, production is activated upon customer’s demand and each accepted job is sold at the selling prince contractually defined with the customer. So, turnover ($TV$) can be easily obtained summing up the selling prices of all the jobs delivered in the considered time interval. Instead, in case of simulation, we suggest compute the turnover of job as in Eq. (12):

12 $$TV_{j} = TR_{j} \cdot \left( {1 + \delta } \right)$$

where $\delta > 0$ is a mark-up percentage applied to the total transformation cost.

The mark-up should cover the indirect industrial costs, a quota of the overhead costs and, eventually, it should generate a profit. This percentage is generally available in the cost accounting system of the firm.

## Performance assessment of a WLC system

To demonstrate the need to introduce the economic model to assess the quality of a WLC system, we reproduced the High-Variability Low Volumes (HVLV) six-machines job-shop, which is typically used as a benchmark in technical literature^68^. To this aim we used Simpy©, an open-source discrete event simulation package, developed in Python 3.7©.

### Job-Shop configuration

Concerning the simulated job-shops:

- All the six machines are equally loaded, with an average utilization rate or 90%;
- They all have constant capacity and full availability;
- Materials and resources are always available; machines can be idle, but starvation never occurs;
- The job shop is ‘pure and randomly routed’. In this regard:
  - Each job is processed by $N$ machines, with $N$ uniformly distributed in the range $\left[ {1, \,6} \right]$;
  - The order in which machines are visited is random, but half of the generated jobs has a ‘directional routing’, while the other half has a ‘purely random routing’. Specifically, machines are labelled with a progressive number (from 1 to 6) and, in case of directional routing, for each couple of successive machines $\left( {i,k} \right)$ in the routing of job $j$, $k$ must be greater than $i$.
  - Rerouting is not allowed, i.e., a machine cannot appear on the routing of job $j$ more than once.
- Processing time $t_{j,m}$ (of each job $j$ on each machine $m$) follows a Gamma Distribution with shape parameter $k = 2$ and scale parameter $\theta = 0.52$. Hence the average processing time on each machine is $\overline{t} = 1.05$ time units and the coefficient of variation is $CV = 0.707$. This choice is typical to reproduce the processing time variability of HVLV job-shops.
- Set-up times must be performed any time a machine processes a new job, but they are not sequence dependent. Hence, set-up times are not explicitly defined, but simply included in the processing times $t_{j,m}$ generated from the Gamma distribution described above;
- Jobs are generated according to a Poisson process, with exponential distributed inter-arrival times. An arrival rate ${ }\lambda = 1.45$ units of time was chosen to obtain, in a purely push-operating system (i.e., jobs never wait in the PSP), the desired 90% utilization rate, for each machine.

### WLC settings

Concerning the WLC used to regulate the job-shop, aiming to investigate how performance and costs change as the operating setting changes, a rather ample set of alternative were tested, as detailed next:

- *Job entry* – All generated jobs are always accepted (i.e., total acceptance rule) and an exogenous due date, imposed by the customer, is assigned to each job upon arrival. The value of the due date is randomly generated following the same logic proposed by ample WLC research. Specifically, as shown in Eq. (13) the due date of job $j$ is obtained adding to the arrival time $AT_{j}$ the expected total processing time $\left( {\overline{t} \cdot N_{j} } \right)$ and a uniformly distributed random allowance $U$.
  13 $$DD_{j} = AT_{j} + U + \left( {N_{j} + n} \right) \cdot \overline{t}$$
  where $n$ is a natural number used to include the extra allowance factor $n\overline{t}$ in the expected processing time $N_{j} \overline{t}$.
- *Job Release*, the following sub setting were used:
  - Jobs in the PSP are considered for possible release every 24 units of time;
  - Norms are defined as upper bound of the workload (expressed in units of expected working time) either using the Total Shop (TSL) load or the load at each machine approach (LEM). So, the decision of releasing a job depends on the value of $W_{t}$ or of $W_{m,t}$ in the first and second case, respectively. Also, in case of load at each machine the upper bound set on $W_{m,t}$ is the same for all machine $m$; a natural choice for a perfectly symmetric job-shop whit equivalent and equally loaded machines.
  - The workload contribution of each entering job is quantified either using the aggregated (AGG) or the corrected aggregated (CAGG) approach, computed as in Eqs. (3) and (5), respectively.
- *Job Dispatching—*Two alternative dispatching rules, namely First In First Out (FIFO) and Planned operation Start Time (PST) are used. The first one is extremely easy (jobs simply queue up) and is the one that minimizes work in process; the second one is very effective in minimizing tardiness or other time related performances^69^. Specifically, when the PST rule is in use, jobs are sorted in ascending order of their planned operation start time. The later one is computed as in Eq. (14):
  14 $$PST_{j} = DD_{j} - \left( {\tilde{N}_{j} + n} \right) \cdot \overline{t}$$

Where $n$ is a natural number and $\tilde{N}_{j} \le N_{j}$ is the number of machines that are yet to be visited by job $j$, so that $\tilde{N}_{j} \overline{t}$ is the remaining expected processing time. In brief, therefore, $PST_{j}$ is an estimate of the net time that is still available to complete the job in time.

Concerning the allowance factors of Eqd. (13) and (14), we consider $U$ as uniformely distributed on the interval $\left[ {0, 30} \right]$ and we set $n = 2$. Both values were empirically defined via simulation, to maximize the percentage of on time deliveries when the job-shop operates in purely push way and the PST rule is used, provided that the percentage of on time deliveries does not drop below 75% (considered as a realistic value) when the FIFO rule is used instead. For the sake of clarity, the settings used for the Job-Shop and for the WLC system are summarized in Table 2.

**Table 2:** Configuration of the simulated Job-shop and WLC settings.

Parameter	Variable	Description
**System’s parameters**		
Number of Machines	No	6 machines
Routing	No	Random length (1 to 6) with random sequence, no re-entrant loops
Processing time	No	Gamma distributed on all machines, with mean 1.05 and variance 0.54
Setup time	No	Included in the operation time
Machines’ utilization	No	90% for all machines
Machines’ availability	No	100% for all machines
Job’s arrival rate	No	Exponentially distributed, $\lambda = 1.45$ job every time units
Exogenous Due Dates	No	Computed as in Eq. (13), average value of 21, net of the entry time
**WLC parameters**		
Jobs Acceptance	No	All jobs are assumed to be feasible, the ‘total acceptance’ criterion is used
Jobs Consideration	No	Discrete timing convention, jobs in the PSP evaluated every 24 units of time
Aggregation of workload measure	Yes	1. Total Shop Load (baseline)
		2. Load at each machine
Workload accounting over time	Yes	1. Aggregated load (baseline)
		2. Corrected aggregated load
Workload control	No	Norms defined as upper bound values
Dispatching rule	Yes	1. FIFO (baseline)
		2. PST

In brief, a total of eight alternatives configurations were obtained, by combining 2 aggregations of workload measure, 2 workload accounting systems and 2 dispatching rules, that is 2 different options for each variable parameter that we considered in our simulation (namely Aggregation of workload measure, Workload accounting over time and Dispatching rule).

Hence, we have 3 variables, each of which can assume 2 alternative levels. Also, if we consider the baseline WLC policies (i.e., total shop load, aggregated approach, FIFO rule) as ‘low factor levels’, and the alternative policies as ‘high factor levels’ (i.e., load at each machine, corrected aggregated approach, PST rule), the design scheme that we tested corresponds to a $2^{3}$ full factorial design with repetitions.

### Performance analysis

For each one of the eight investigated configurations, we recorded the performance observed for different levels of the norms. More precisely, starting from an almost infinite level, corresponding to a purely push operating system, we progressively constrained the system by reducing the norms’ level proceeding with step of 1%. This procedure was interrupted as soon as the observed throughput dropped below 1.44 [jobs per time unit], that is the lower end of the 95% confidence interval of the throughput of the unconstrained system. In this way the full domain of the solutions ensuring the maximum throughput is obtained, with the last generating one corresponding to the condition that minimizes work in process and queues. For each investigated point we performed a simulation trial of 10 runs, each of 3650 units of time. A warm-up period of 1200 units of time, enough to reach the steady state of the system, was also included in each simulation run. These simulation parameters are consistent with those generally used in case of job-shop simulations and provide stable results in an adequate amount of time.

**Figure 1:**
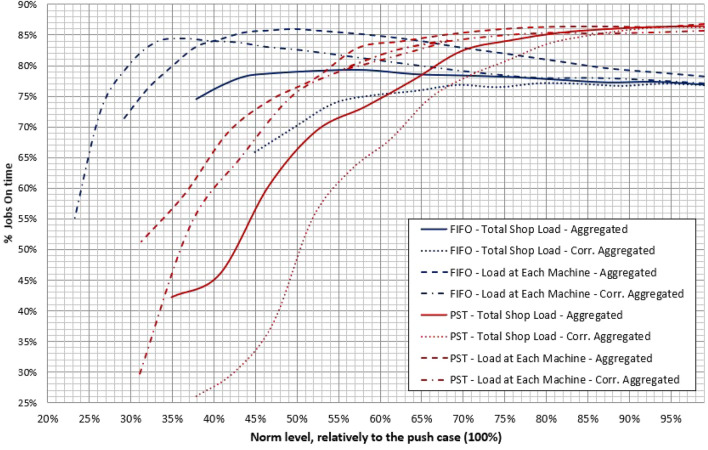
Trend of the percentage of tardy job of eight WLC configurations.

An example is given in Fig. 1, where the percentage of jobs delivered in time (performance measure) is plotted on the $y$ axis, versus the norm level plotted on the ${ }x$ axis. Note that the latter one is expressed as a percentage of the unconstrained case, that is a value equal to 100% correspond to an unconstrained system (i.e., norms are never violated, and jobs are always released upon arrival, without pending in the PSP).

A first consideration concerns norm levels, marking a significant difference between FIFO rule, performing much better in terms of on time jobs for low values of the norms, that is a relatively starved system, and PST rule, which overperforms FIFO at higher norm levels (i.e., roughly above 55%).

Figure 1 is also in line with WLC literature to confirm that better on time performances are obtained with the load at each machine approach and with PST dispatching rule to sort jobs both in the pre-shop pool and in the shop floor. However, in this case, a clear maximum does not exist, as the red curves flatten out for high values of the norms, when the WLC system approximates a purely push-operating job shop. In other words, in terms of the percentage of on time orders, the performance of a purely push system based on the PST rule does not deviates much from that of a WLC system. The system, in fact, is undersaturated (90% utilization level) and so, even if jobs are pushed in production, queues do not diverge, and the use of an appropriate rule (such as the PST) is enough to assure a good performance. Also note that the red curves fall very quickly when work in process is reduced moving from right to left, that is when moving away from the purely push condition. Hence, improvements achievable in terms of WIP reduction are limited. Conversely, as the blue curves demonstrate, the FIFO rule coupled with the load at each machine approach ensures a high reduction in WIP at the expense of a modest deterioration of the percentage of on time deliveries. In this case, in fact, there is a clear maximum point, which is located in the left-hand side of the graph.

So, as anticipated in “Performance assessment of a WLC system” section, there is a clear trade off among different solutions, and choosing one of them based on a single criterion could be somehow misleading. This concept is expressed in an even clearer way by Table 3 where, for each investigated WLC configuration, the average value of the following performance measures is given:

- *Gross Throughput Time—****GTT***, the overall time spent by a job in the system both as an order pending in the PSP, or as an actual job being processed on the shop floor.
- *Shop-Floor Throughput Time—****SFTT***, the overall time spent by a job in the shop floor (also known as production Lead Time).
- *Work In Process—****WIP***, the amount of jobs processed in the shop floor, measured as units of remaining working time.
- *Percentage of jobs in time—****% IN TIME***, the number of jobs completed and delivered before their due date over the total number of accepted jobs.
- *Tardiness—****Tar***, the difference between the completion (or delivery) time $C_{j}$ and the due date $DD_{j}$, if the difference is positive, zero otherwise., i.e., $T_{j} = {\text{max}}\left\{ {0,\left( {C_{j} - DD_{j} } \right)} \right\}$.
- *Earliness—****Ear***, the difference between the due date and the completion time, if the difference is positive, zero otherwise., i.e., $E_{j} = {\text{max}}\left\{ {0,\left( {DD_{j} - C_{j} } \right)} \right\}$.

**Table 3:**
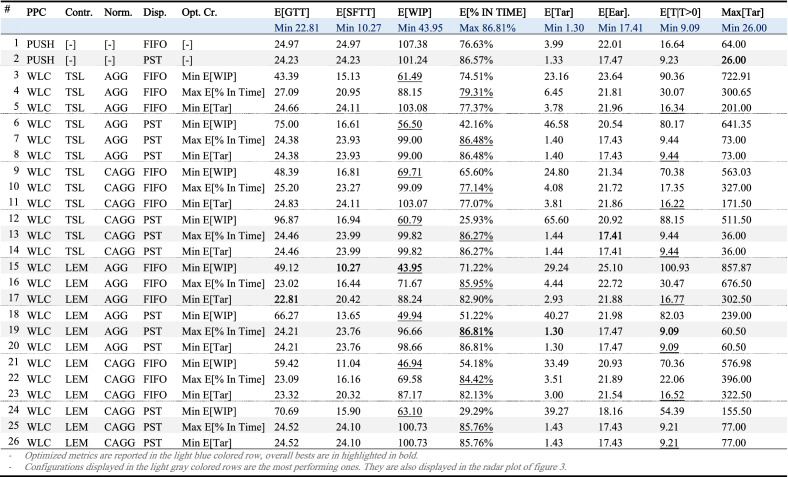
Performance measures of the investigate WLC configurations.

Optimized metrics are reported in the light blue colored row, overall bests are in highlighted in bold, and local best (i.e., best results for each specific WLC configuration).

Configurations displayed in the light gray colored rows are the most performing ones. They are also displayed in the radar plot of Fig. 3.

As anticipated, all these metrics are evaluated in average terms, with the exception of the tardiness, for which we have also provided its maximum value and its conditional expectation ${\mathbb{E}}\left[ {T\left| T \right\rangle 0} \right]$, that is the mean taken on jobs with non-zero delay only. We also note that, since both the earliness and the tardiness were provided, we decided not to include the earliness $E_{j} = \left( {C_{j} - DD_{j} } \right)$ in Table 3. For the sake of completeness and of clarity the overall best is displayed immediately underneath the corresponding metric, and it is also highlighted in light blue color.

Lastly, we note that, for each considered configuration, norms have been fined tuned relatively to three alternative objective functions that, as indicated in the fifth column of the table are: (i) the minimum level of WIP, (ii) the maximum percentage of on time delivery, and (iii) the minimum tardiness. For this reason, the table is organized in blocks of three rows, one for each configuration and objective function. Note that sometimes, the values of two adjacent rows may be identical (as for rows 13 and 14); in these cases, although fine tuning was made with respect to different performance parameters, the same configuration (in terms of norm levels) was found.

As a benchmark, the same parameters have also been computed for the purely push-operating system equipped with FIFO and PST rules, respectively.

The WLC configuration based on the load at each machine approach, with corrected aggregated workload and PST dispatching rule, is the one that seems to perform best, as it optimizes three out of eight metrics. Yet, a clear dominant solution does not exist and, even more surprisingly, the minimum maximum tardiness is provided by the push operating system with PST dispatching rule.

**Figure 2:**
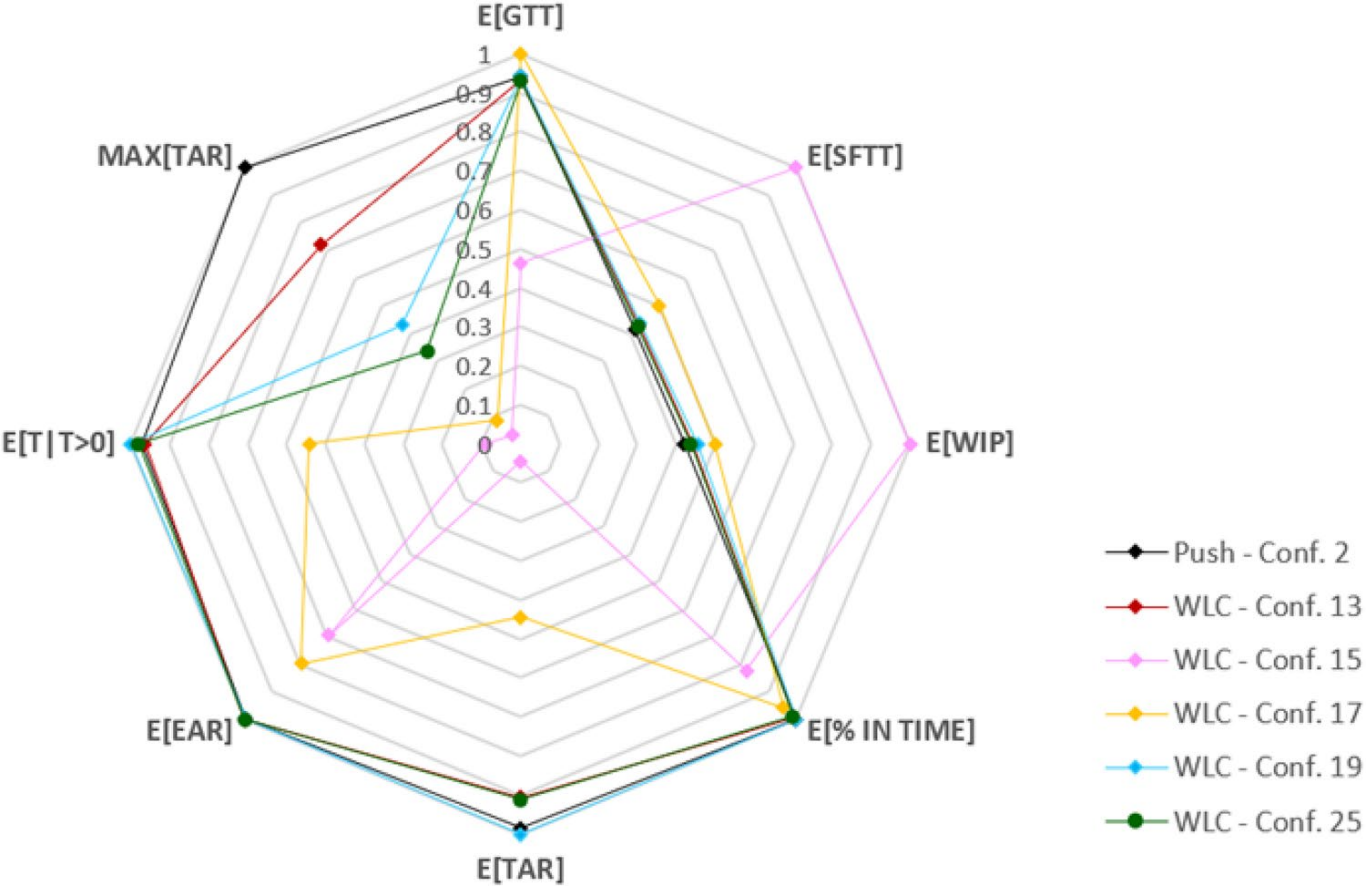
Radar plot of the observed performance indicators.

An overall view of this fact is finally given by the radar chart of Fig. 2, where obtained performances are shown in relative terms. Specifically, we included in the chart all the configurations that achieved the absolute maximum for at least one performance. These configurations correspond to the rows 2, 13, 15, 17 and 19 that are highlighted in grey in Table 3. Note that configuration 2 is the push case, while configurations 13, 19 and 25 are the ones that optimize time related performances. As already noted, they all have a trend very similar to that of the push case, except for the maximum tardiness, that is reduced. We note that Fig. 2 also includes configuration 25, a WLC configuration that achieved good performances, on average, on all metrics.

Even this plot clearly shows that, if WLC is fine tuned to minimize lateness related objectives the difference between WLC and Push are marginal, and perhaps not enough to justify WLC implementation in the industry. Besides, relevant improvements can be obtained focusing on WIP reduction, especially when dispatching rules less prone to the prioritization of urgent orders are used. In this case, however, while it is still possible to keep the percentage of jobs in time at a fairly good level, the number of outliers with a very high tardiness increases significantly (see for example row 15 and 17 of Table 3). These are the jobs with high workload that wait a long time in the PSP before being released and that, once in the shop floor, although being late, are not prioritized and go through all the queues.

It is thus clear that selecting and fine tuning a WLC configuration based on a single metric is a myopic approach, that may lead to a sub-optimal solution that does not exploits the full potentiality of WLC. Hence, in the following section we will discuss how the use of the economic approach herein proposed can be effective to solve this problem.

## Performance assessment in monetary terms

### Parameters’ setting

To introduce adequate costs in the simulated scenario, the following parameters were used:

- Cost of direct material ${\mathcal{M}}$ uniformly distributed on the interval $\left[ {10, 30} \right]$.
- Processing cost per unit of time $p_{m} = 17.75$, for each machine $m$.
- Stock holding coefficient $k_{h} = 0.12\%$.
- Penalties coefficient $$k_{p} = 3k_{h} = 0.36\%$$.
- Mark Up coefficient $\delta = 17.5\%$.

We also note that:

- All direct materials are supposed to be added to a job at its first operation, so Eq. (9) can be used.
- Since setup time were included in the processing time of each job, we also assumed that the quota of the setup cost per job is included in the processing cost per unit of time $p_{m}$.
- We limited the maximum penalty to the contractual selling price $P_{j}$, that is $PN_{j} = {\text{min}}\left\{ {P_{j} ,\left( {k_{p} P_{j} T_{j} } \right)} \right\}$, where $T_{j}$ is the tardiness. Hence, if the tardiness is so high that the penalty equals the contractual price the income is null (as for a lost sale) and there is a loss equal to all costs incurred.

Using these settings, in the baseline case (i.e., the push operating system with FIFO dispatching rule) the average selling price is 100, the average total cost is 90, for an expected operating revenue, which we will simply refer to as revenue per job, of 10 units for each job sold. We note that the system we proposed could be easily adapted to different cost and income settings.

### Obtained results

**Figure 3:**
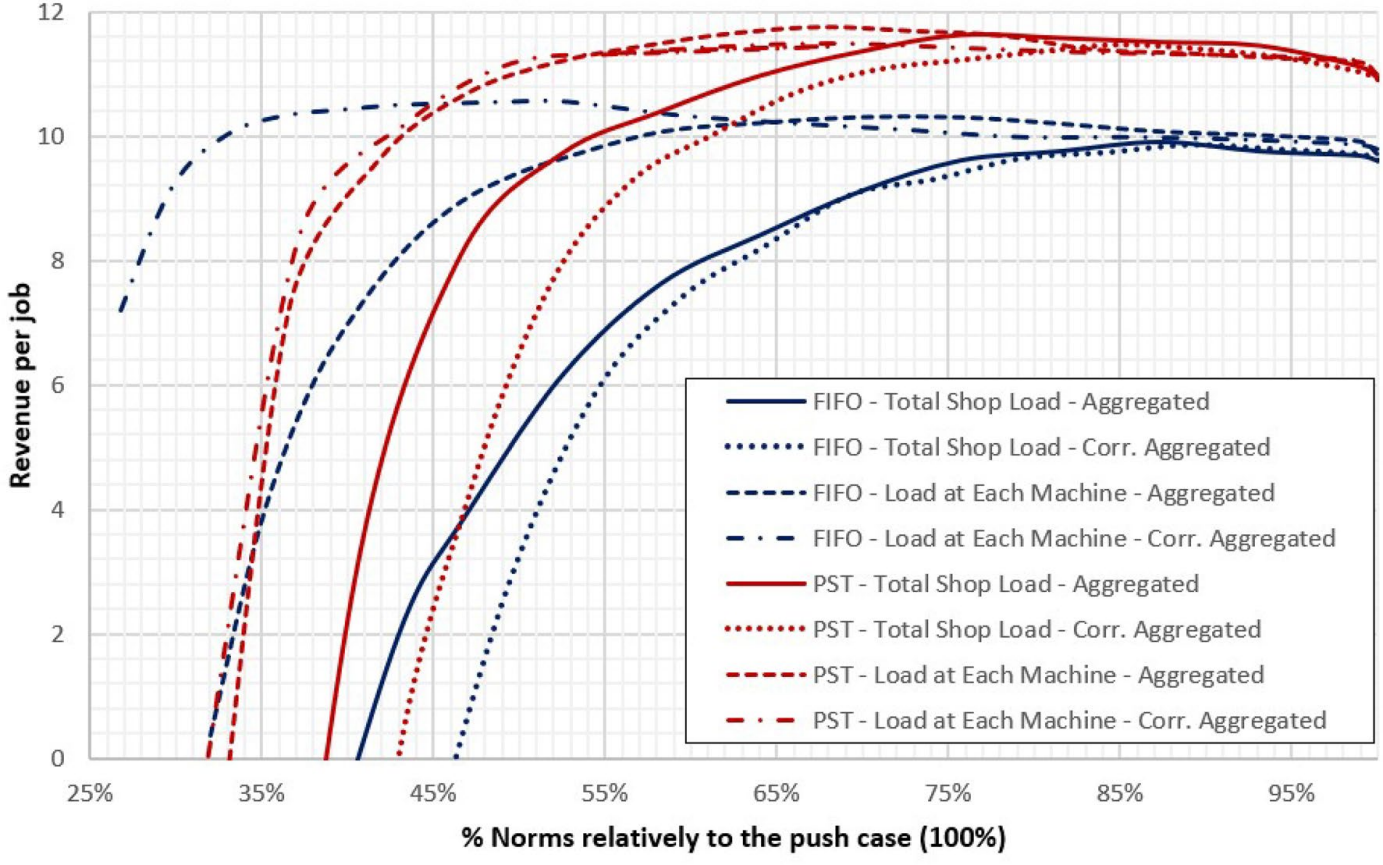
Trend of revenues per job of eight WLC configurations.

The trend of the average revenue per job (on the $y$ axis) versus the norm level (on the $x$ axis) is shown in Fig. 3, for all eight investigated configurations (see also Fig. 1 and Table 2). We note at first that the PST norm significantly outperform FIFO in terms of Revenue per Job. Also, WLC configurations with Aggregated load perform slightly better than those with Corrected aggregated load, and the Aggregated load at Each machine achieves the highest value of Revenue per job. However, we believe important to note that, although the curves, and specifically PST curves (i.e. the red-brown ones), are rather flat for high values of the norms, a global maximum always exists as all curves are convex. This fact means that all WLC configuration we tested achieved a maximum in the Revenue per job function for some value of the norm below 100%, i.e. in a non-push condition.

To assess the impact that our the operational parameters we selected have on costs and incomes, we performed a preliminary 4-ways ANOVA. Specifically, we used the revenue per job as response variable and (i) Norm level, (ii) Aggregation of workload measure, (iii) Workload accounting over time and (iv) Dispatching rule as factors. The analysis confirmed all the hypotheses and indeed all factors and interactions were significant with a $p$-value lower than 0.005, with the only exception of the Workload accounting over time criterium, i.e. aggregated load vs. corrected aggregated load. For this reason, we excluded it from the analysis from now on. For a more detailed explanation on this point, please refer to Online Appendix A.

This fact is confirmed by the one-way ANOVA of Tables 4 and 5, that reveals that the points of maximum revenue per job of the alternative configurations differ significantly, in statistical terms. Note that, since the effect of the Workload accounting over time resulted non-significant in the preliminary 4-ways ANOVA, the analysis was limited to four WLC alternatives and the push system was taken as benchmark.

**Table 4:** One-ways ANOVA summary results.

Groups	Count	Sum	Mean	Variance
PUSH—FIFO	10	96.505	9.650	0.5517
WLC—FIFO—TSL	10	96.875	9.687	0.5400
WLC—FIFO—LEM	10	103.333	10.333	0.1716
PUSH—PST	10	109.552	10.955	0.0711
WLC—PST—TSL	10	117.388	11.738	0.0186
WLC—PST—LEM	10	113.806	11.380	0.0742

*TSL* Total Shop Load, *LEM* Load at Each Machine.

**Table 5:** One-ways ANOVA results.

Source of variance	Sum of squares	Deg. of freedom	Mean of squares	F value	*p*-value	F crit
Between groups	38.340	5	7.6681	32.228	4.608E−15	2.386
In groups	12.848	54	0.2379			
Total	51.188	59				

As Table 4 reports, revenues per job clearly differ when the PST rule is used instead of the FIFO one. This fact was also confirmed by a Tukey–Kramer post-hoc test (at level $\alpha = 0.1)$; a synthesis of the pairwise comparison is provided in Table 6, which also highlights a significant difference between the optimal WLC configuration and the corresponding push condition (reported with bold character in Table 6).

**Table 6:** Tukey–Kramer test.

Config. #1	Config. #2	Mean diff	Critical value	Significant
WLC—PST—TSL	PUSH—FIFO	2.088	0.751	Yes
WLC—PST—TSL	WLC—FIFO—TSL	2.051	0.751	Yes
WLC—PST—TSL	WLC—FIFO—LEM	1.405	0.751	Yes
WLC—PST—TSL	PUSH—PST	**0.783**	**0.751**	Yes
WLC—PST—TSL	WLC—PST—LEM	0.358	0.751	No

It is interesting to note that the WLC configuration that maximizes the expected Revenue per job is the same one that maximized the percentage of on time deliveries (as we found in “Performance assessment of a WLC system”section), namely Aggregation of workload method: Load at each machine; Workload accounting over time: Aggregated; and Dispatching rule: PST. Yet, as it is shown by Table 7, the operating point that maximises the value of Revenue per job clearly differs from the one that maximizes the percentage of jobs delivered in time. Specifically, as it is shown in Table 7, which displays the norm level at each machine, either in units of time, or as a percentage relative to the norm of the push case, to maximise revenues in our specific scenario it is advisable to reduce the level of the norms to reduce WIP and stockholding costs. This improvement is large enough to positively balances the extra costs related to a small deterioration of the percentage of on time deliveries, that are necessarily incurred when the norms’ level is reduced. It is also worth noting that the difference in expected revenue, obtained at these two alternative operating points, is statistically significant with a *p*-value of a one-tailed T-test $\ll 0.01$. On the contrary, the expected revenue of the WLC system maximizing the percentage of jobs delivered in time does not significantly differ from that of the benchmark push-system, with a *p*-value > 0.1. This is another clear indication of the opportunity to fine-tune a WLC system considering the expected Revenue per job, rather than a single performance measure.

**Table 7:** Summary results.

PPC	Optimiz. criterium	NORM	%NORM (%)	E[GTT]	E[SFTT]	E[WIP]	E[% IN TIME] (%)	E[Tar]	E[Ear]	E[T|T > 0]	Max[TAR]	E[Rev]
PUSH	[-]	40	100	24.23	24.23	101.24	86.57	1.33	17.47	9.23	26.00	10.97
WLC (LEM-AGG-PST)	Min E[% In Time]	38	95	24.21	23.76	96.66	86.81	1.30	17.47	9.09	60.50	11.12
WLC (LEM-AGG-PST)	Max E[Revenue]	26	65	24.89	21.11	79.20	85.12	1.59	17.29	9.83	80.40	11.74

### Sensitivity analysis

**Figure 4:**
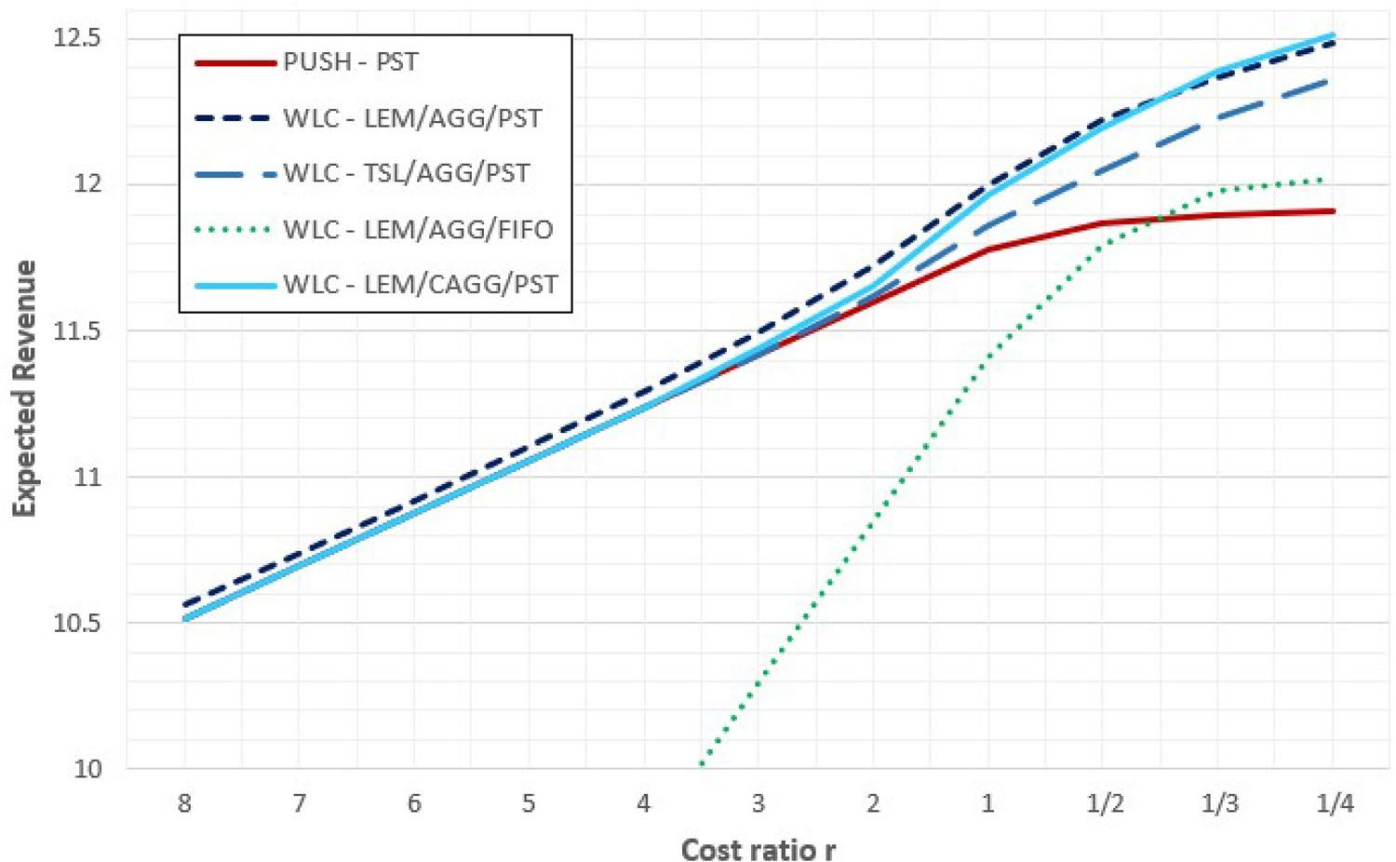
Sensitivity analysis.

Clearly, the results we discussed above depend on the cost structure under analysis and, most of all, on the values of the coefficients $k_{h}$ and $k_{p}$ used to estimate the stockholding and the penalties cost, respectively. As above discussed, in fact, these costs directly reflect antithetical operating performances (mainly WIP level and tardiness) for which a tradeoff must be achieved. Hence, for a further insight, we repeated the optimization analysis for different values of the cost ratio $r = \left( {\frac{{k_{p} }}{{k_{h} }}} \right)$, by fixing $k_{h}$ at its original value of 0.12% and by progressively reducing $k_{h}$ from $8k_{h}$ to $0.25k_{h}$. Obtained results are graphically summarized in Fig. 4, which shows the maximum expected revenue that can achieved using the most effective WLC configurations, each one fine-tuned for each level of the investigated cost ratio $r$.

As it can be seen, the WLC configurations based on the aggregate approach, load for every machine and the PST rules is almost ever the dominant solutional and the positive gap, increases as the ratio $r$ decreases. As already noted, indeed, this configuration is the one that tends to optimize the trade-off between low WIP and short and stable gross throughput time, and this fact becomes particularly relevant as the stockholding costs increase relative to the penalty ones. The only exception is the WLC configuration based on corrected aggregate approach, load for every machine and the PST rules, that becomes the best option for very high levels of the stockholding cost (the indifference point is found around $r = 2/5$). The use of the corrected aggregate approach, in fact, is more precise, but also more selective and binding; hence, relatively to the aggregate approach, it is more performant in terms of WIP, but less in terms of percentage of tardy job. In term of cost, this condition becomes valuables only when $r$ is small. For the same reason for low levels $r$, even the use of the FIFO policies becomes quite performing, as it outperforms the benchmark push approach, yet it remains lower than the other two configurations. Conversely, the economical gap among the benchmark push approach and the WLC policies based on the PST rule becomes almost inappreciable when $r$ is big.

## Conclusions, managerial implications, and future works

This paper deals with WLC, an hybrid push–pull PPC system that has seen a great deal of attention in the last decades. Focusing on performance assessment of rule-based WLC systems, the paper shows that setting the norms and other operating parameters by considering the percentage of tardy job as the only optimization criterion is generally sub-optimal. To get the most out of WLC, in fact, and to achieve benefits that could justify the required initial investment, practitioners should look for a trade-off between WIP reduction and due date compliance. To simplify this multi-criteria optimization problem, we propose to homogenize alternative performance criteria, even conflicting ones, by translating them in monetary terms. To this aim, we developed a general economic model to properly consider the economic valorization of the operating performances regarding a WLC system, namely the Revenue per job. It is the authors’ opinion, in fact, that such a trade-off might only be identified and analyzed by means of an economic model, which enables the comparison of different system performances. Also, without an economic model that justifies the benefits of WLC, companies, and especially SMEs, will not be supported to implement ‘off-the-shelves’ WLC systems. We do believe, instead, that an adequate and realistic economic calculation of WLC benefits might help SMEs to decide for its implementation. Industrial sectors that could benefit from the approach we propose in this study are sub-contract machining shops, producers of mechanical components (e.g. for the automotive industry) and producers of electronic components (e.g. wafer fabrication).

The model has been finally tested via simulation and validated through a comprehensive statistical analysis. What emerged from the analysis is the fact that the economic optimum point (i.e. the maximum expected revenue per job) hardly ever coincides with the point that optimizes a single performance measure. The difference is even more prominent in case of high stock holding costs, comparable to those related to penalties. We note that the existence of these economic optimum points is calculated in a simulated system taken from the mainstream of WLC research, whose parameters and values have been used with profit by previous studies aiming at minimizing shop floor throughput times and percentages of tardy jobs in WLC systems. Thus, we believe that the existence of said optimum values in the Revenue per job function of such a simulated system justifies the need to look for it from a broader, and more general, perspective. Also, we reported how the specific performances of different WLC configurations might impact on the economic method we propose, and we reported a sensitivity analysis that, starting from the preliminary observations of^20,22^, shows the results of a continuous variation of the cost ratio *r*, and it allows the extension of our WLC evaluation approach to different sectors and industries. As an example, if we recall the above-mentioned sectors, a low *r* value is expected in companies producing electronic components, where penalty costs are higher than holding costs. Conversely, a high *r* value is typical of sub-contract machining shops and of producers of mechanical components, where WIP value and holding costs are higher. Thus, these sectors could use the model we propose to better understand cost savings or other benefits expected from WLC adoption.

In general, with respect to the WLC configurations we investigated, the best economic performance can be achieved with a WLC system based on aggregate load, load at each machine, and PST dispatching rule. Yet, a WLC system based on the corrected aggregate approach becomes preferable for lower levels of the cost ratio $r = \left( {\frac{{k_{h} }}{{k_{p} }}} \right)$. Indeed, in this condition the additional WIP reduction that can be achieved is enough to offset the unavoidable worsening of delivery times. In this regard, it has to be said that our model underestimates the benefits related to WIP reduction. Less WIP, indeed, reduces errors (as visual management becomes easier), and assures other savings related to improved tidiness and reduced occupied space that could be dedicated to other activities. Since these savings have not yet been captured by our model, it is likely that the corrected aggregated approach becomes advantageous also at higher value of the ratio $r$. For the same reason, advantages of WLC over the benchmark push-oriented job shop might become evident also for values of $r$ lower than 3, where, at the moment, the economic difference between WLC and push is very small. In this respect, we finally note that we limited the analysis to a rather unsaturated system with an average utilization rate of 90%. This decision, which is in line with the mainstream of WLC literature, suggests that the economic advantages achievable by applying WLC to almost saturated systems (i.e., utilization rate of 95% or higher, at least for certain amounts of time), with respect to push-operated systems, could be bigger than what we report. In such conditions, in fact, push systems will greatly diverge from hybrid or pull ones, in terms of queues and shopfloor throughput time. All these issues could be further investigated in subsequent works. Also, aligned with research mainstream on WLC, our model considers only machines as constrained resources. In some situations, this might not be the case, as human resources could be equally critical bottlenecks (e.g. manual assembly station or testing benches). We note that, in these cases, the general approach we followed still holds true, but some improvements are needed, and scientific research on this topic is still scarce. Developing a broader WLC approach capable of operating with a combination of human and machine resources, which takes into consideration the peculiarities of each one of them, could definitely be a topic of future research. Another important aspect that is currently undervalued in most WLC studies is the preliminary identification and mapping, and the subsequent management of critical constrained resources. As we mentioned above, the focus is (almost) only on machines, which are often considered identical in terms of utilization and availability rates. Thus, much WLC research does not consider stable or shifting bottlenecks. Therefore, considering an early identification and management of critical constrained resources, be them human or machines, could bridge the gap among WLC and other PPC systems (e.g. theory of constraints and drum-buffer-rope), and extend the impact of WLC on flexibility, by covering other aspects of flexibility, such as labor and material handling flexibility.

To conclude, we provide a last remark concerning the FIFO policy. As shown in the present paper, although this rule assures significant advantages in terms of WIP, it is always penalizing in economic terms. As discussed in “Performance analysis”section, the problem is mainly related to a minority of jobs requiring very high processing times. The fact is that these outliers wait for very long time in the PSP, before being admitted in the shop floor; also, once in the system, although already late, they are forced to queuing up because of the FIFO policy. Hence, incurred penalties are very high and detrimental in terms of revenue per job. To limit this issue, it could be wise to devise an acceptance policy that rejects new orders with a heavy workload, anytime the workload in the system approaches norms’ level, or to incorporate some other expediting corrections to the order release policy that could improve the system. As an example, only regular job should be sorted using the FIFO rule, whereas jobs that have been waiting for long in the PSP should be prioritized. The economic evaluation of this approach could be the topic of a future work.

## Supplementary Information

Supplementary Information.

## Data Availability

The datasets generated and/or analysed during the current study are available in the ‘**wlc-economic-valorization**’ repository, available at the current web link: https://github.com/dmezzogori/wlc-economic-valorization. Please, note that the repository might be amended and improved at a later stage, upon paper’s acceptance or by answering to reviewers comments.
