# Supplementary material for: A new perspective on Workload Control by measuring operating performances through an economic valorization
Source: Sci Rep. 2022 Aug 26;12:14599. doi: 10.1038/s41598-022-17968-5 (PMC9418317; doi:10.1038/s41598-022-17968-5)
Supplement: Supplementary file 1 — Supplementary Information. [file 41598_2022_17968_MOESM1_ESM.docx]

**Appendix A.**

To get an initial indication of the relative impact the operational parameters have on costs and incomes, a 4-ways ANOVA was performed, using the revenue per job as response variable and the following four factors as independent variables:

- Factor 1 – Norm Level evaluated at six levels {95%, 85%, 75%, 65%, 55%, 45%}, where 100% is the norm that does not restrict at all the job release phase.
- Factor 2 - Job release strategy either based on a Total Shop Load (level 1) or on Load at Each Machine (level 2).
- Factor 3 – Workload accounting either based on the aggregate (level 1) or the corrected aggregate (level 2) approach.
- Factor 4 – Dispatching rule either FIFO (level 1) or PST (level 2).

We also note that a total of ten repetitions were made for each combination of the levels and that, as suggested by Thürer and Stevenson (2016), the ANOVA was based on a block design, with the norm level as the blocking factor. In other words, the six levels of the norm were treated as different systems, as this approach allows one to capture the main effect of the workload norm and both the main and interaction effects of the other three factors. Performing this analysis, a R-Squared of 0.521 and an Adjusted R-Squared of 0.509 were obtained. Also, the $p$ value of the overall corrected model is null, a fact that confirms that revenues change by modifying the WLC configuration. The detailed results of the ANOVA are reported in Table A1.

As it can be seen, all factors except the workload accounting strategy have a clear effect on costs; similarly, only the interaction between the release approach and the dispatching rule is significant, while all the other ones are not. Probably, this can be explained as it follows. The corrected aggregate approach allows a finer control and, therefore it shifts the optimal operating point to a lower level of WIP. At this point, however, the percentage of tardy jobs is slightly bigger than that achievable with the aggregated technique that, being less restrictive, allows a greater release of orders into the system. The two factors offset each other in terms of cost, making the choice almost indifferent.

**Table A1.** 4-ways ANOVA results

| **Source of Variance** | **Sum of Squares** | **Deg. Of. Freed.** | **F-Ratio** | **p-value** |
| --- | --- | --- | --- | --- |
| *Intercept* | 13727.683 | 1 | 3333.554 | 0 |
| *Norm Level* | 1024.969 | 5 | 49.779 | 0 |
| *Release Strategy* | 450.9663 | 1 | 109.510 | 0 |
| *Workload Accounting* | 12.57183 | 1 | 3.053 | 0.081 |
| *Dispatching Rule* | 500.9520 | 1 | 121.648 | 0 |
| *Release × Workload* | 25.88853 | 1 | 6.286 | 0.0125 |
| *Release × Dispatching* | 70.07254 | 1 | 17.016 | 4.39E-05 |
| *Workload × Dispatching* | 1.984883 | 1 | 0.481 | 0.487 |
| *Release × Workload × Dispatching* | 2.810446 | 1 | 0.682 | 0.41 |
| *Residual* | 1923.120 | 467 |  |  |
